# Functional brain imaging interventions for radiation therapy planning in patients with glioblastoma: a systematic review

**DOI:** 10.1186/s13014-022-02146-8

**Published:** 2022-11-12

**Authors:** John T Ryan, Masao Nakayama, Ian Gleeson, Liam Mannion, Moshi Geso, Jennifer Kelly, Sweet Ping Ng, Nicholas Hardcastle

**Affiliations:** 1 Department of Medical Imaging and Radiation Sciences, Monash University, Clayton, Melbourne, Australia; 2 Medical Radiations Department, School of Health and Biomedical Sciences, STEM College, RMIT University Bundoora, Melbourne, Australia; 3 Division of Radiation Oncology, Kobe University Graduate School of Medicine, 7-5-2 Kusunokicho, Chuou-ku, Kobe, Japan; 4 Cancer Research UK RadNet Cambridge, Medical Physics, NHS Foundation Trust, Addenbrookes Hospital, Cambridge, CB2 0QQ UK; 5 Division of Midwifery and Radiography, School of Health Sciences, University of London, Northampton Square, London, UK; 6 Department of Radiation Oncology, Olivia Newton-John Cancer Wellness and Research Centre, 145 Studley Rd, Heidelberg, Melbourne, Australia; 7 Department of Physical Sciences, Peter MacCallum Cancer Centre, 305 Grattan St, Melbourne, Australia

**Keywords:** Systematic review, Functional imaging, Glioblastoma, Radiotherapy planning

## Abstract

**Rationale:**

This systematic review aims to synthesise the outcomes of different strategies of incorporating functional biological markers in the radiation therapy plans of patients with glioblastoma to support clinicians and further research.

**Methods:**

The systematic review protocol was registered on PROSPERO (CRD42021221021). A structured search for publications was performed following PRISMA guidelines. Quality assessment was performed using the Newcastle–Ottawa Scale. Study characteristics, intervention methodology and outcomes were extracted using Covidence. Data analysis focused on radiation therapy target volumes, toxicity, dose distributions, recurrence and survival mapped to functional image-guided radiotherapy interventions.

**Results:**

There were 5733 citations screened, with 53 citations (n = 32 studies) meeting review criteria. Studies compared standard radiation therapy planning volumes with functional image-derived volumes (n = 20 studies), treated radiation therapy volumes with recurrences (n = 15 studies), the impact on current standard target delineations (n = 9 studies), treated functional volumes and survival (n = 8 studies), functionally guided dose escalation (n = 8 studies), radiomics (n = 4 studies) and optimal organ at risk sparing (n = 3 studies). The approaches to target outlining and dose escalation were heterogeneous. The analysis indicated an improvement in median overall survival of over two months compared with a historical control group. Simultaneous-integrated-boost dose escalation of 72–76 Gy in 30 fractions appeared to have an acceptable toxicity profile when delivered with inverse planning to a volume smaller than 100 cm$^3$.

**Conclusion:**

There was significant heterogeneity between the approaches taken by different study groups when implementing functional image-guided radiotherapy. It is recommended that functional imaging data be incorporated into the gross tumour volume with appropriate technology-specific margins used to create the clinical target volume when designing radiation therapy plans for patients with glioblastoma.

**Supplementary Information:**

The online version contains supplementary material available at 10.1186/s13014-022-02146-8.

## Background

There is a clinical and economic need for improved outcomes for patients diagnosed with glioblastoma. Globally, over 300,000 primary brain and central nervous system cancers were diagnosed in 2020, with glioblastoma accounting for 48.6% of primary brain and central nervous system cancers in the United States (US) [1, 2]. The outcomes from care are poor; a patient’s relative survival post-diagnoses is under 7% at 5-years with current best practice [3]. In Australia, primary brain cancers account for more disability adjusted life-years lost per patient than any other adult cancer [4]. Further, brain cancer diagnoses have the largest financial cost of all cancers [5].

The World Health Organisation has classified four categories of diffuse gliomas: adult-type diffuse gliomas, pediatric-type diffuse high-grade gliomas, pediatric-type diffuse low-grade gliomas and circumscribed astrocytic gliomas with glioblastoma grouped with isocitrate dehydrogenase (IDH)-wildtype as adult-type diffuse gliomas [6]. The current standard treatment for glioblastoma consists of maximum safe surgical excision, followed by concurrent chemo-radiotherapy (typically 60 Gy/30 fractions or 40 Gy/15 fractions in elderly patients) with concurrent temozolomide (TMZ), followed by adjuvant TMZ [7]. Standard treatment has remained relatively unchanged in the last 17 years [8, 9]. Patient outcomes with standard treatment include a median overall survival of 14.6 months, a median progression-free survival of 6.9 months, a 12 months overall survival of 61.1% and a 12 months progression-free survival of 26.9% [9, 10]. Interestingly, the control arms of more recent randomised control trials which follow the Stupp et al. 2005 [11] guidelines report a median survival of approximately 20 months, most likely as a result of treatment refinement [11, 12].

Radiation therapy (RT) treatment quality has been shown to influence patient outcomes with target delineation identified as one of the largest variables in the treatment process [13, 14]. Currently, RT management of patients with glioblastoma is fragmented by the existence of variable target delineation guidelines which are based on magnetic resonance imaging (MRI) and computed tomography (CT) [15, 16]. This fragmentation in practice results in a significant difference in the outlined clinical target volume (CTV), for instance, in a study by Kumar et al. the CTV varied from 246 to 436 cm$^3$ depending on the guidelines followed [17]. The guidelines documenting the acceptability of positron emission tomography (PET) scanning to support the RT planning of patients with gliomas have recently changed with PET scanning now more likely [18]. There is also increasing evidence to investigate dose-escalated RT in patients with glioblastoma, particularly in subgroups that do not receive TMZ or those who are known to have unmethylated DNA repair enzyme O-6-methylguanine-DNA methyltransferase (MGMT) [19]. Therefore, RT quality and patient outcomes are highly dependent on target voluming guidelines and the dose fractionation regimes used.

The main concern with dose escalation or increases in target volume size is an increased risk of side effects such as radiation necrosis [20]. This has prompted research to investigate biologically-derived target volumes and RT boosts to improve the specificity and sensitivity of the treated volumes and facilitate safe dose escalation [19, 21, 22]. Currently, 75% of patients receiving standard care for glioblastoma recur local to the high dose RT volume [23]. This highlights the need to improve both the local control and the sensitivity of target localisation, as nearly 100% of glioblastoma patients progress with current practice. Key to this goal, is capturing detailed recurrence information with geometric reference to the standard International Commission on Radiotherapy Units (ICRU) volumes and pre-treatment imaging, as this can be used to quantify the success of target contouring.

Functional imaging modalities have the advantage of being able to identify biochemical changes that often predate, or are distinct from, anatomical changes [24, 25]. A diverse range of functional imaging biological markers, including MRI, magnetic resonance spectroscopic imaging (MRSI) and nuclear medicine (NM), hold promise in this new era of functional image-guided radiotherapy (FIGR) [22, 25]. Additionally, the novel disciplines of radiomics and dosiomics, will have an increasingly synergistic role alongside functional imaging in the management of RT patients [26]. However, there is an evidence gap to support the development of guidelines to realise the benefits of FIGR. Therefore, the aim of this systematic review is to synthesise strategies and outcomes of functional imaging for RT planning in patients with glioblastoma. Outcomes related to RT target volumes, dose distributions, toxicity, recurrences and survival will be synthesised to support clinicians and research.

## Method

This systematic review was conducted in accordance with the Preferred Reporting Items for Systematic Reviews and Meta-Analyses (PRISMA) statement [27]. The review was prospectively registered on PROSPERO (CRD42021221021) [28]. The search strategy was conducted in consultation with a research librarian.

### Search strategy

Literature searches were conducted in January 2021 in PubMed, CINAHL, Scopus, Cochrane Library, EMBASE, Web of Science and AMED. Three concepts were used to guide searches: functional imaging, glioblastoma multiforme and radiotherapy planning. Indexing thesaurus, keywords, MeSH terms, CINAHL headings, Indexed terms, Emtree terms, and synonyms were used, as relevant. Searches were restricted to texts available in English and published from January 2011 to January 2021, due to the rapid development in this field. A full overview of search terms, dates and boolean operations used for each database is available in Additional file 1.

### Eligibility criteria

Eligibility criteria were defined according to participants, intervention, comparator, outcome and study type (PICOS framework). Participants and comparators were patients with primary glioblastoma undergoing external beam radiotherapy with a curative intent. Participants were 18 years of age or over. Mixed cohorts (i.e., glioblastoma and other high grade glioma) had to report separate outcomes for patients with glioblastoma to be eligible. Journal articles and published conference abstracts were included. Review articles and unpublished grey literature were excluded. Purely explorative articles (i.e., radiomics studies that were not trialled in patients with glioblastoma) were excluded.

### Study selection

**Fig. 1:**
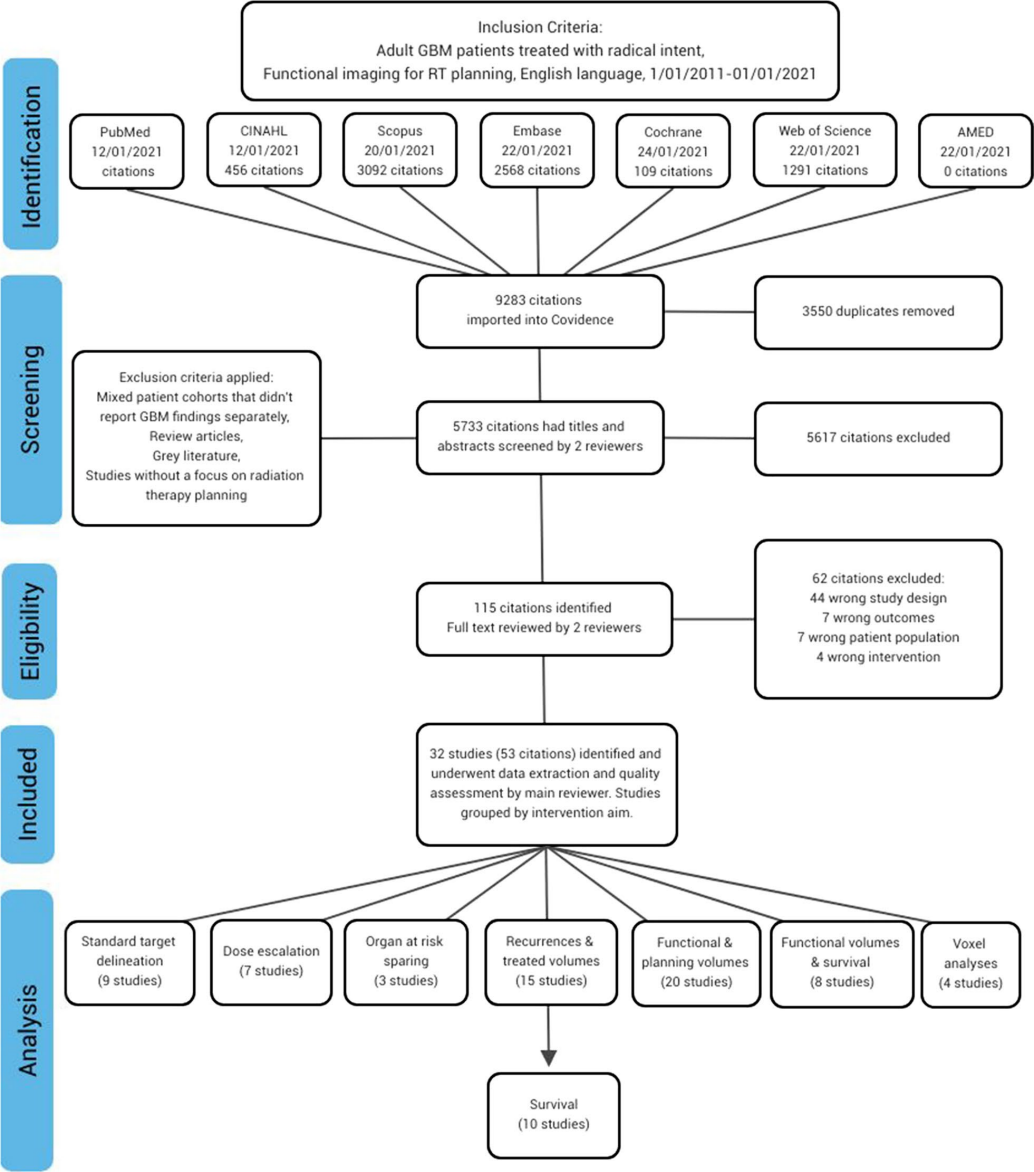
Flow diagram (adapted from the PRISMA guidelines) demonstrates the screening and evaluation process

Duplicates were removed in Covidence [29]. Title and abstract screening against eligibility criteria was completed by two reviewers (from JR, MN, IG or LM). Conflicts were resolved by an independent reviewer (NH or JR). Eligibility of full text articles was determined by two reviewers (from JR, MN or IG) reading each paper in full. Conflicts were resolved by consultation. Data extraction was completed by JR. Figure 1 provides an overview of the screening, exclusion rationale and data extraction.

### Data extraction and management

Citations were grouped according to study cohort. Study characteristics were extracted using a Covidence template and the Template for Intervention Description and Replication (TIDieR) checklist [30]. Data were extracted according to study ID, title, author, information source, location, funding, conflicts, study aim, study design, participant description, sample size, attrition, confounding variables, intervention and TIDieR components. Study quality including biases for journal articles was assessed using the Newcastle–Ottawa Scale (NOS) assessment tool (Table 1) [31].

**Table 1:** Study characteristics

Study name	Location	Citations	Data		Par n	Study type					Q	Aim						
			Conf. abstract	Journal	Participants	RCT Phase 1	RCT Phase 2	RCT Phase 3	Prosp. cohort	Retro. cohort	Study Quality	Standard target delineation	Dose escalation	Organs at risk sparing	Recurrences &treated volumes	Functional volumes& planning volumes	Functional volumes& survival	Radiomic features
Niyazi	Europe	[32]	$\checkmark$		17					$\checkmark$						$\checkmark$		
		[33]		$\checkmark$	17					$\checkmark$	9				$\checkmark$	$\checkmark$		
Piroth	Europe	[34]		$\checkmark$	44				$\checkmark$		9		$\checkmark$				$\checkmark$	
		[35]	$\checkmark$		22	$\checkmark$	$\checkmark$						$\checkmark$				$\checkmark$	
		[36]		$\checkmark$	22		$\checkmark$						$\checkmark$				$\checkmark$	
		[37]		$\checkmark$	13		$\checkmark$								$\checkmark$			
Laouiti	Europe	[38]	$\checkmark$		12	$\checkmark$	$\checkmark$				N	$\checkmark$	$\checkmark$					
Miwa	Asia	[39]	$\checkmark$		51	$\checkmark$	$\checkmark$				N	$\checkmark$	$\checkmark$					
Munck Af	Europe	[40]		$\checkmark$	54				$\checkmark$			$\checkmark$				$\checkmark$		
Rosenschold		[41]		$\checkmark$	190					$\checkmark$	9	$\checkmark$				$\checkmark$	$\checkmark$	
Lundemann	Europe	[42]	$\checkmark$		66					$\checkmark$		$\checkmark$			$\checkmark$	$\checkmark$		
		[43]		$\checkmark$	66					$\checkmark$		$\checkmark$			$\checkmark$	$\checkmark$		
		[44]		$\checkmark$	14				$\checkmark$		9				$\checkmark$			$\checkmark$
Poulsen	Europe	[45]	$\checkmark$		146					$\checkmark$		$\checkmark$					$\checkmark$	
		[46]		$\checkmark$	146					$\checkmark$	7	$\checkmark$					$\checkmark$	
Harat	Europe	[47]		$\checkmark$	29					$\checkmark$	9				$\checkmark$	$\checkmark$		
Hayes	Aus/NZ	[48]		$\checkmark$	24					$\checkmark$	8	$\checkmark$			$\checkmark$	$\checkmark$		
Fleischmann	Europe	[49]		$\checkmark$	36					$\checkmark$	9				$\checkmark$	$\checkmark$		
Matsuo	Asia	[50]		$\checkmark$	32				$\checkmark$		9					$\checkmark$		
		[51]	$\checkmark$		32				$\checkmark$							$\checkmark$		
Vigil	Europe	[52]	$\checkmark$		40					$\checkmark$			$\checkmark$		$\checkmark$	$\checkmark$	$\checkmark$	
		[53]	$\checkmark$		50					$\checkmark$	9		$\checkmark$		$\checkmark$	$\checkmark$	$\checkmark$	
Hirata	Asia	[54]	$\checkmark$		25					$\checkmark$					$\checkmark$	$\checkmark$	$\checkmark$	
		[55]		$\checkmark$	25					$\checkmark$	9				$\checkmark$	$\checkmark$	$\checkmark$	
Christensen	USA	[56]	$\checkmark$		6					$\checkmark$					$\checkmark$	$\checkmark$		
		[57]		$\checkmark$	11					$\checkmark$	7				$\checkmark$	$\checkmark$		
Kosztyla	Canada	[58]		$\checkmark$	19				$\checkmark$		6				$\checkmark$	$\checkmark$		
Brinkmann	USA	[59]	$\checkmark$		32		$\checkmark$									$\checkmark$		
		[60]	$\checkmark$		77		$\checkmark$					$\checkmark$	$\checkmark$					
		[61]	$\checkmark$		75		$\checkmark$				N	$\checkmark$	$\checkmark$				$\checkmark$	
Windisch	Europe	[62]	$\checkmark$		13				$\checkmark$							$\checkmark$		
		[63]		$\checkmark$	13				$\checkmark$		7					$\checkmark$		
Munshi	Asia	[64]		$\checkmark$	24					$\checkmark$	7					$\checkmark$		
Wahl	USA	[65]		$\checkmark$	52					$\checkmark$	9				$\checkmark$	$\checkmark$	$\checkmark$	
Kim	USA	[66]	$\checkmark$		20		$\checkmark$						$\checkmark$				$\checkmark$	
		[67]		$\checkmark$	12		$\checkmark$						$\checkmark$				$\checkmark$	
		[68]	$\checkmark$		26		$\checkmark$				9		$\checkmark$				$\checkmark$	
Berberat	Europe	[69]		$\checkmark$	13					$\checkmark$	8				$\checkmark$	$\checkmark$		
Wang	Asia	[70]		$\checkmark$	20					$\checkmark$	8			$\checkmark$				
Zhang	USA	[71]	$\checkmark$		100				$\checkmark$		N	$\checkmark$						
Altabella	Europe	[72]		$\checkmark$	19					$\checkmark$	9			$\checkmark$				
Peeken	Europe	[73]		$\checkmark$	33					$\checkmark$	9				$\checkmark$	$\checkmark$		$\checkmark$
Morin	USA	[74]	$\checkmark$		31					$\checkmark$	N	$\checkmark$			$\checkmark$	$\checkmark$		
Anwar	USA	[75]		$\checkmark$	24					$\checkmark$	9				$\checkmark$			$\checkmark$
Opposits	Europe	[76]		$\checkmark$	13	$\checkmark$					9			$\checkmark$				
Laprie	Europe	[77]	$\checkmark$		16					$\checkmark$			$\checkmark$					
		[78]		$\checkmark$	16					$\checkmark$	9		$\checkmark$					
		[79]	$\checkmark$		165			$\checkmark$					$\checkmark$					
		[80]		$\checkmark$	180			$\checkmark$			9		$\checkmark$					
Lopez	India/USA	[81]		$\checkmark$	17					$\checkmark$	9					$\checkmark$		$\checkmark$
Mellon	USA	[82]	$\checkmark$		30	$\checkmark$							$\checkmark$					
		[83]	$\checkmark$		30	$\checkmark$					N		$\checkmark$					
Gurbani	USA	[84]		$\checkmark$	1				$\checkmark$		9					$\checkmark$		

Study name	Intervention																	
	F18-FET PET	C11-MET PET	C11-AMT PET	F18-FDOPA PET	F18-FDG PET	Ga68-FAP PET	DCE-MRI	DTI-MRI	DW-MRI	PWI-MRI	BOLD-MRI	MRSI	Boost					
Niyazi	$\checkmark$																	
	$$\checkmark$$																	
Piroth	$\checkmark$												$\checkmark$					
	$\checkmark$												$\checkmark$					
	$\checkmark$												$\checkmark$					
	$\checkmark$												$\checkmark$					
Laouiti	$\checkmark$												$\checkmark$					
Miwa	$\checkmark$												$\checkmark$					
Munck Af	$\checkmark$																	
Rosenschold	$\checkmark$																	
Lundemann	$\checkmark$																	
	$$\checkmark$$																	
	$\checkmark$				$\checkmark$		$\checkmark$		$\checkmark$									
Poulsen	$\checkmark$																	
	$$\checkmark$$																	
Harat	$\checkmark$																	
Hayes	$\checkmark$																	
Fleischmann	$\checkmark$																	
Matsuo		$\checkmark$																
		$$\checkmark$$																
Vigil		$\checkmark$											$\checkmark$					
		$\checkmark$											$\checkmark$					
Hirata		$\checkmark$			$\checkmark$													
		$\checkmark$			$\checkmark$													
Christensen			$\checkmark$															
			$$\checkmark$$															
Kosztyla				$\checkmark$														
Brinkmann				$\checkmark$									$\checkmark$					
				$\checkmark$									$\checkmark$					
				$\checkmark$									$\checkmark$					
Windisch						$\checkmark$												
						$$\checkmark$$												
Munshi							$\checkmark$											
Wahl							$\checkmark$		$\checkmark$									
Kim							$\checkmark$		$\checkmark$				$\checkmark$					
							$\checkmark$		$\checkmark$				$\checkmark$					
							$\checkmark$		$\checkmark$				$\checkmark$					
Berberat								$\checkmark$										
Wang								$\checkmark$			$\checkmark$							
Zhang								$\checkmark$										
Altabella								$\checkmark$										
Peeken								$\checkmark$										
Morin									$\checkmark$									
Anwar									$\checkmark$	$\checkmark$		$\checkmark$						
Opposits											$\checkmark$							
Laprie												$\checkmark$	$\checkmark$					
												$\checkmark$	$\checkmark$					
												$$\checkmark$$						
												$\checkmark$	$\checkmark$					
Lopez												$\checkmark$						
Mellon												$\checkmark$	$\checkmark$					
												$\checkmark$	$\checkmark$					
Gurbani												$\checkmark$	$\checkmark$					

*RCT* Randomised controlled trial, *Par* Participants, *Q* Quality (Score 1–9 or N = Non applicable, A low score is indicative of some issue with the study quality), $\checkmark$ = Present in citation

Studies were grouped according to their aims, including improvement of standard target delineation, facilitation of dose escalation, improvement of organ at risk (OAR) sparing, mapping recurrence and treated volumes, mapping functional and planning volumes, mapping functional volumes and survival, and carrying out voxel/radiomics analyses (Fig. 1 and Table 1). Study outcomes and confounders are captured in Tables 2, 3 and 4. A study quality score of less than nine in Table 1 indicates an issue with data reliability in an individual study, such as reporting in a short time frame post-intervention or not reporting select patient data that may have biased the results.

### Study synthesis

The tables present a snapshot of the included studies (Table 1) and examine clinically relevant RT planning and associated patient outcomes (Tables 2, 3 and 4). An analysis of outcomes relating to median overall survival, median progression-free survival, 12-month overall survival percentage, and 12-month progression-free survival was completed (Table 5). In Table 5 studies were weighted based on their population number and the summed averages compared to a historical control. Data were otherwise reported narratively.

## Results

A total of 32 studies (53 citations) met inclusion criteria (Table 1 and Fig. 1) . TIDieR components were inconsistently reported [25–76]. Information about who carried out the intervention and their expertise was lacking. Few studies used a prospective control group.

### Included study overview

Included studies consisted of journal articles (n = 25) and conference presentations (n = 7). There were 20 retrospective cohort studies, eight prospective cohort studies, seven Phase I or Phase II trials and one Phase III randomised controlled trial.

Studies were grouped into seven categories comparing standard RT planning volumes with functional-derived volumes (n = 20), treated RT volumes with recurrences (n = 15), functional imaging into standard target delineations (n = 9), functional imaging volumes and survival (n = 8), functional imaging to guide dose escalation (n = 8), voxel or radiomics analyses (n = 4) and OAR sparing based on functional information (n = 3). There were 13 different technological interventions directly related to functional imaging, categorised according to NM, MRI, MRSI and FIGR boost. Dose escalation based on FIGR was implemented in eight studies using NM agents (n = 5), MRSI (n = 2) and MRI (n = 1).

### Study outcomes

Twenty studies included outcomes on RT target volumes, 12 reported toxicity with a FIGR intervention, 10 reported survival with a FIGR intervention and 15 reported recurrence. Two of the 32 studies were not included in Tables 2, 3, 4 and 5 for analysis. Matsuo et al. [50, 51] conducted a volume comparison study and reported on the sensitivity and specificity of the CTV with different gross tumour volume (GTV) to CTV expansion margins. Lopez and colleagues [81] established a framework of co-dependencies between MRI, MRSI and radiotherapy planning volumes using radiomics.

#### Target volume size

Target volume size for RT planning volumes was recorded in Table 2. Target volume reporting between studies was varied, with 11 studies reporting median volumes and four studies reporting mean volumes. The GTV that incorporated functional and anatomical imaging was larger than the anatomical GTV in all studies. The intra-study variation in standard CTV size was greater than the inter-study variation in CTV size that incorporated functional and anatomical imaging. Target volume creation with functional imaging was heterogeneous. The most common approach was to incorporate the functional imaging data directly into the CTV or the planning target volume (PTV) without a specific uncertainty margin and by using standard GTV to CTV margin recipes. The CTV and PTV change with combined imaging was methodology dependent and varied between a 27% increase to a 50% decrease. The dose escalated boost volume was less than 100 cm$^3$.

**Table 2:** Target volume outcomes

Study name	Citations	Functional imaging agent	Intervention aim							Treatment		
			Improve standard RTtarget delineation	Facilitate doseescalation	Facilitate organ at risk sparing	Map recurrence & RT treated volumes	Map functional volumes& RT planning volumes	Map treated functionalvolumes & survival	Map radiomic features& RT volumes	Standard treatment	Standard targetvolumes changed	Dose escalation
Niyazi	[32, 33]	F18-FET PET					$\checkmark$			$\checkmark$		
Piroth	[34–37]	F18-FET PET		$\checkmark$		$\checkmark$		$\checkmark$			$\checkmark$	$\checkmark$
Munck Af Rosenschold	[40, 41]	F18-FET PET	$\checkmark$				$\checkmark$	$\checkmark$			$\checkmark$	
Lundemann	[42–44]	F18-FET PET	$\checkmark$			$\checkmark$	$\checkmark$		$\checkmark$		$\checkmark$	
Poulsen	[45, 46]	F18-FET PET	$\checkmark$					$\checkmark$			$\checkmark$	
Harat	[47]	F18-FET PET				$\checkmark$	$\checkmark$			$\checkmark$		
Hayes	[48]	F18-FET PET	$\checkmark$			$\checkmark$	$\checkmark$				$\checkmark$	
Fleischmann	[49]	F18-FET PET				$\checkmark$	$\checkmark$			$\checkmark$		
Hirata	[54, 55]	C11-MET & F18-FDG PET				$\checkmark$	$\checkmark$	$\checkmark$		$\checkmark$		
Christensen	[57]	C11-AMT PET				$\checkmark$	$\checkmark$			$\checkmark$		
Kosztyla	[58]	F18-FDOPA PET				$\checkmark$	$\checkmark$			$\checkmark$		
Windisch	[62, 63]	FAP PET					$\checkmark$			$\checkmark$		
Munshi	[64]	DCE MRI					$\checkmark$			$\checkmark$		
Wahl	[65]	DW & DCE MRI				$\checkmark$	$\checkmark$	$\checkmark$		$\checkmark$		
Berberat	[69]	DTI MRI				$\checkmark$	$\checkmark$			$\checkmark$		
Altabella	[72]	DTI MRI			$\checkmark$					$\checkmark$		
Peeken	[73]	DTI MRI				$\checkmark$	$\checkmark$		$\checkmark$	$\checkmark$		
Morin	[74]	DW MRI	$\checkmark$			$\checkmark$	$\checkmark$			$\checkmark$		
Laprie	[77–80]	MRSI		$\checkmark$								$\checkmark$
Gurbani	[84]	MRSI				$\checkmark$	$\checkmark$					$\checkmark$

Study name	RT target volume details											
	GTV-S (cm$^3$)	GTV-F (cm$^3$)	GTV-S+F (cm$^3$)	CTV-S (cm$^3$)	CTV-F (cm$^3$)	CTV-S+F (cm$^3$)	PTV-S (cm$^3$)	PTV-F (cm$^3$)	PTV-S+F (cm$^3$)	CTV Target change (%)	PTV Target change (%)	Boost Volume (cm$^3$)
Niyazi	*x* 34.1	*x* 43.9	*x* 48	*x* 224.5	*x* 240.3	*x* 286.8	*x* 343.5	*x* 356.5	*x* 416.5	*x* +27.7	*x* +21.1	
Piroth	$\bar{x}$ 5.2	$\bar{x}$ 14.3					$\bar{x}$ 219.5					$\bar{x}$ 14.3
Munck Af Rosenschold	*x* 37	*x* 19.8	*x* 38.2	$\bar{x}$ 275	$\bar{x}$ 290	$\bar{x}$ 300				$\bar{x}$ +9.1		
Lundemann	*x* 33.8	*x* 21.4	*x* 41.6									
Poulsen		*x* 21.8										
Harat		*x* 30.4										
Hayes				*x* 83.6		*x* 94.7				*x* +13.3		
Fleischmann	*x* 14.0	*x* 19.8	*x* 30.8				*x* 297.8		*x* 271.3		*x* $-$8.9	
Hirata	*x* 9.9	*x* 60.4	*x* 60.4									
Christensen	$\bar{x}$ 50.2	$\bar{x}$ 48.9	$\bar{x}$ 67.6									
Kosztyla	$\bar{x}$ 30.8	$\bar{x}$ 58.6	$\bar{x}$ 63.5	$\bar{x}$ 249	$\bar{x}$ 306	$\bar{x}$ 312	$\bar{x}$ 369	$\bar{x}$ 437	$\bar{x}$ 445	$\bar{x}$ +25.3	$\bar{x}$ +20.6	
Windisch	*x* 33.8	*x* 23.8 - 49.6	*x* 60.8				*x* 271					
Munshi	*x* 40.8	*x* 17.2	*x* 46.3	*x* 200.1		*x* 221.0	*x* 258		*x* 286	*x* +10.4	*x* +10.8	
Wahl	$\bar{x}$ 37.8	$\bar{x}$ 21.4										
Berberat										$-$50	$-$15	
Altabella							$\bar{x}$ 543					
Peeken	*x* 23.3	*x* 5.9		*x* 207.2$^\alpha$ - 276.8$^\beta$		*x* 240.9				*x* -13.0 - +16.3		
Morin										*x* -26.7		
Laprie									*x* 307.8			*x* 97.63
Gurbani		50.6										$\le$ 65

*S* Standard contouring, *F* Functional imaging used, S+F= combined, *x* = Median, $\bar{x}$ = Mean, $\alpha$ = EORTC guidelines, $\beta$ = RTOG guidelines

#### Dose distribution, toxicity and survival

Toxicity and survival metrics associated with the RT treatment process are presented (Table 3). Dose escalation was used in eight studies. The dose-escalated fractionation schedules had an equivalent 2 Gy doses (EQD$_2$) ranging between 74.4 and 104.8 Gy, with a given alpha/beta ratio of 10. Only one study used an EQD$_2$ > 79.4 Gy [39]. Toxicity in the dose-escalated studies with an EQD$_2$ of $\leqslant$ 79.4 Gy were well tolerated, when delivered as a simultaneous integrated boost, with 60 Gy in 30 fractions prescribed to the standard PTV. A limited boost size $\leqslant$ 65 cm$^3$ was recommended in one study [85].

**Table 3:** Survival and toxicity outcomes

Study name	Citations	Functional imaging agent	Intervention aim							Treatment		
			Improve standard RTtarget delineation	Facilitate doseescalation	Facilitate organ at risk sparing	Map recurrence & RT treated volumes	Map functional volumes& RT planning volumes	Map treated functionalvolumes & survival	Map radiomic features& RT volumes	Standard targetvolumes changed	Dose escalation	Functional OAR sparing
Piroth	[34–37]	F18-FET PET		$\checkmark$		$\checkmark$		$\checkmark$		$\checkmark$	$\checkmark$	
Laouiti	[38]	F18-FET PET	$\checkmark$								$\checkmark$	
Miwa	[39]	F18-FET PET	$\checkmark$								$\checkmark$	
Munck Af Rosenschold	[40, 41]	F18-FET PET	$\checkmark$				$\checkmark$	$\checkmark$		$\checkmark$		
Lundemann	[42–44]	F18-FET PET	$\checkmark$			$\checkmark$	$\checkmark$		$\checkmark$	$\checkmark$		
Poulsen	[45, 46]	F18-FET PET	$\checkmark$					$\checkmark$		$\checkmark$		
Vigil	[52, 53]	C11-MET PET		$\checkmark$		$\checkmark$	$\checkmark$	$\checkmark$		$\checkmark$	$\checkmark$	
Brinkmann	[59, 61, 61]	F18-FDOPA PET	$\checkmark$	$\checkmark$			$\checkmark$	$\checkmark$		$\checkmark$	$\checkmark$	
Kim	[66, 68, 86]	DW MRI & DCE MRI		$\checkmark$				$\checkmark$			$\checkmark$	
Wang	[70]	DTI MRI & BOLD MRI			$\checkmark$							$\checkmark$
Zhang	[71]	DTT MRI	$\checkmark$							$\checkmark$		
Altabella	[72]	DTI MRI					$\checkmark$					$\checkmark$
Morin	[74]	DW MRI	$\checkmark$			$\checkmark$	$\checkmark$			$\checkmark$		
Opposits	[76]	BOLD MRI			$\checkmark$					$\checkmark$		
Laprie	[77–80]	MRSI		$\checkmark$							$\checkmark$	
Mellon	[82]	MRSI		$\checkmark$							$\checkmark$	

Study name	Max dose			Dose fractionation (Gy / fractions)	Toxicity/OAR dose metric	Survival						
	BED$_{2}$ (Gy)	BED$_{10}$ (Gy)	EQD$_2$ (Gy)			Median OS Mths	Median PFS Mths	12 Mths OS %	12 Mths PFS %			
Piroth	158.4	89.3	74.4	72/30	No grade 3 or 4 toxicity	14.8	7.8	63.6	25.4			
Laouiti	158.4	89.3	74.4	72/30	Maximum grade CTCAE acute toxicity was 1 (median), range 0-2			38.4	28.6			
Miwa	357	125.8	104.8	68/8		20	13	71.2	52.6			
Munck Af Rosenschold	120	72	60	60/30	Plan OAR (brain, brain stem...) receive less dose in PET-image guided-VMAT plans	15	6					
Lundemann	120	72	60	60/30		20.7	11.7					
Poulsen	120	72	60	60/30		16.5	6.5					
Vigil	Not reported but escalated					20	6.7					
Brinkmann	172.3	95.3	79.4	76/30	Grade 3 CNS necrosis was noted in 3 patients (4.4%), 1 patient (1.5%) with pre-existing vision dysfunction had Grade 4 optic nerve dysfunction	16	8.7					
Kim	168.8	93.8	78.1	75/30	Side effects were similar in incidence to standard therapy	20		90				
Wang	120	72	60	60/30	Plan OAR (white matter tracks) received less dose in functionally optimised plan							
Zhang	120	72	60	60/30	Thecognition dysfunction was mild and the radiation-induced brain oedema was mild to moderate	17.6		76	66			
Altabella	120	72	60	60/30	Plan OAR (white matter structures) received less dose in DTI-optimised plans							
Morin	120	72	60	60/30	Plan OAR (hippocampus) received less dose in DW-optimised plans							
Opposits	120	72	60	60/30	Reduced dose in the functionally active areas of the brain							
Laprie	158.4	89.3	74.4	72/30	SIB plan OAR (brainstem and brain) received lower doses than CRT plans							
Mellon	168.8	93.8	78.1	75/30	No observed serious adverse events							

* BED*$_2$ Biological effective dose (alpha/beta = 2), *BED*$_{10}$ Biological effective dose (alpha/beta = 10), *EQD*$_2$ Equivalent dose in 2 Gy (alpha/beta = 10), *OS* Overall survival, *PFS* Progression free survival, *Mths* Months, *CTCAE* Common terminology criteria for adverse events, *OAR* Organ at risk, *SIB* Simultaneous -integrated boost, *CRT* Conformal radiation therapy

#### Recurrence patterns

Fifteen studies reported on recurrence outcomes (Table 4). These were broken down into three categories: studies that involved a retrospective comparison with a standard approach, studies that used functional imaging to derive the target volumes for treatment and studies that outlined extra target volumes with associated dose escalation. Table 4 indicates the relative location of a recurrence as a proportion of the total number of recurrences. Consistency was limited in the reporting of recurrence patterns and their location in relation to imaging and ICRU volumes. There were a higher proportion of recurrences central to the high therapeutic dose volume when functional and anatomical imaging were combined to outline the target.

**Table 4:** Recurrence outcomes

Study name	Citations	Functional imaging agent	Intervention aim							Treatment		
			Improve standard RTtarget delineation	Facilitate doseescalation	Facilitate organ at risk sparing	Map recurrence & RT treated volumes	Map functional volumes& RT planning volumes	Map treated functionalvolumes & survival	Map radiomic features& RT volumes	Standard treatment	Standard targetvolumes changed	Dose escalation
Niyazi	[32, 33]	F18-FET PET				$\checkmark$	$\checkmark$			$\checkmark$		
Piroth	[34–37]	F18-FET PET		$\checkmark$		$\checkmark$		$\checkmark$			$\checkmark$	$\checkmark$
Lundemann	[42–44]	F18-FET PET	$\checkmark$			$\checkmark$	$\checkmark$		$\checkmark$		$\checkmark$	
Harat	[47]	F18-FET PET				$\checkmark$	$\checkmark$			$\checkmark$		
Hayes	[48]	F18-FET PET	$\checkmark$			$\checkmark$	$\checkmark$				$\checkmark$	
Fleischmann	[49]	F18-FET PET				$\checkmark$	$\checkmark$			$\checkmark$		
Vigil	[52, 53]	C11-MET PET		$\checkmark$		$\checkmark$	$\checkmark$	$\checkmark$			$\checkmark$	$\checkmark$
Hirata	[54, 55]	C11 MET & F18-FDG PET				$\checkmark$	$\checkmark$	$\checkmark$		$\checkmark$		
Christensen	[56, 57]	C11-AMT PET				$\checkmark$	$\checkmark$			$\checkmark$		
Kosztyla	[58]	F18-FDOPA PET				$\checkmark$	$\checkmark$			$\checkmark$		
Wahl	[65]	DW & DCE MRI				$\checkmark$	$\checkmark$	$\checkmark$		$\checkmark$		
Berberat	[69]	DTI MRI				$\checkmark$	$\checkmark$			$\checkmark$		
Peeken	[73]	DTI MRI				$\checkmark$	$\checkmark$		$\checkmark$	$\checkmark$		
Morin	[74]	DW MRI	$\checkmark$			$\checkmark$	$\checkmark$			$\checkmark$		
Anwar	[75]	DWI & PWI MRI, & MRSI				$\checkmark$			$\checkmark$	$\checkmark$		

Study name	Max dose			Dose fractionation (Gy/fractions)	Recurrence pattern						Recurrence comments	
	BED$_{2}$ (Gy)	BED$_{10}$ (Gy)	EQD$_2$ (Gy)		Central-S %	Central-S+F %	Marginal-S %	Marginal-S+F %	Distal-S %	Distal-S+F %		
Niyazi	120	72	60	60/30	50	50	25	33.3	25	16.7		
Piroth	158.4	89.3	74.4	72/30		78		16.6		5.3		
Lundemann	120	72	60	60/30		82		12		6		
Harat	120	72	60	60/30		69		8		23	Recurrences were significantly more frequent in the areas with marginal therapeutic dose (57-59.9 Gy)	
Hayes	120	72	60	60/30							High agreeance between T2 FLAIR and FET activity and recurrence location	
Fleischmann	120	72	60	60/30	83.3	88.9	11.1	5.6	5.6	5.6		
Vigil	Not reported but escalated										MRI recurrence volume matched the highest uptake area of GTV MET in 30/40 patients	
Hirata	120	72	60	60/30							Decoupling score volume was predictive of recurrence	
Christensen	120	72	60	60/30	58	72	14	14	28	14		
Kosztyla	120	72	60	60/30	72	86	14	0	14	14		
Wahl	120	72	60	60/30							Functional and anatomical combined volume was twice as likely to include recurrence than either independent volume alone	
Berberat	120	72	60	60/30	0-50	0-50	0-50	0-50	50	50		
Peeken	120	72	60	60/30		100						
Morin	120	72	60	60/30	80	100	0-20	0	0-20	0		
Anwar	120	72	60	60/30	0-77		0-77		23		25% of recurrence voxels resided outside the traditional 2 cm treatment boundary. 75% of voxels within the 2 cm treatment margin did not progress	

* BED*$_2$ Biological effective dose (alpha/beta = 2), *BED*$_{10}$ Biological effective dose (alpha/beta = 10), *EQD*$_2$ Equivalent dose in 2 Gy (alpha/beta = 10), *S* Standard imaging, S + F = Standard and functional imaging combined

#### Analysis of the effect of functional imaging on survival

Survival analysis (n = 10 studies, N = 686 participants) indicated improved survival outcomes with FIGR compared to the 2005 Stupp et al. trial (Table 5) [9]. Dose escalation, guided by functional imaging (n = 6 studies, N = 235 participants) further increased median overall survival and median progression-free survival, of these studies, only the Brinkmann study used a local current control group [59–61].

**Table 5:** Survival analyses when functional imaging incorporated in radiation therapy planning

Study name	Citation	N (patients)	Intervention	Median OS (months)	Median PFS (months)	12 Mths OS %	12 Mths PFS %
Piroth	[34–37]	22	F18-FET & 72 Gy/30 fractions boost	14.8 (95% CI 12.7–16.0)	7.8 (95% CI 5.1–10.5)	63.6	25.4
Laouiti	[38]	12	F18-FET & 72 Gy/30 fractions boost			38.4	28.6
Miwa	[39]	51	F18-FET & 68 Gy/8 fractions boost	20	13	71.2	52.6
Munck Af Rosenschold	[40, 41]	190	F18-FET	15 (range 2.04–48.0)	6 (range 2.04–48.0)		
Lundemann	[42–44]	15	F18-FET	20.74 (range 9.53–25.12)	11.7 (range 4.06–17.91)		
Poulsen	[45, 46]	146	F18-FET	16.5	6.5		
Vigil	[52, 53]	50	C11-MET & boost	20	6.7		
Brinkmann	[59, 61, 61]	75	F18-FDOPA & 76 Gy/30 fractions boost	16	8.7		
Kim	[66, 68, 86]	25	DW & DCE & 75 Gy/30 fractions boost	20 (95% CI 14-NA )		90 (95% CI 0.8–1.0)	
Zhang	[71]	100	DTT	17.6 (range 6–42)		76	66
**Stupp et al.**	[9]	**573**	**Historical control**	**14.6 (95% CI 13.2–16.8)**	**6.9 (95% CI 5.8–8.2)**	**61.1**	**26.9**
**Analysis all**		**686**	**Functional imaging**	**16.88**	**7.44**	**70.86**	**55.05**
**Analysis boost**		**235**	**Functional imaging & boost**	**18.52**	**9.31**	**73.05**	**42.17**

Bold indicate that it is different type of data than the data directly above

*N* Number of, *OS* Overall survival, *PFS* Progression free survival, *Mths* Months, *NA* Non applicable, Analyses formula = (n1(f1) + n2(f2 )+... n5(f5))/n total

## Discussion

To the authors knowledge this is the first systematic review of functional image-guided RT interventions in glioblastoma patients. Recent findings to support FIGR in glioblastoma patients have been shared and indicated FIGR can improve patient survival outcomes in certain patient cohorts [87, 88].

### Standard target delineation

The classification of the target volumes for patients with glioblastoma tumours is not static, that is, it varies on the time-point, resources and local expertise available. Most studies using FIGR edited the CTV or PTV directly with historical margin recipes (Table 2). This is problematic as interventions that produce new functional imaging volumes have their own uncertainties that are inherent to the technologies and processes used. Study teams were typically cautious about incorporating functional imaging directly in the GTV, perhaps due to concerns about the final target volume size. However, an increased target size can be avoided with technology-specific GTV-CTV margin recipes (Table 3).

### Dose escalation

Functional image-guided radiotherapy provides a mechanism to give a more personalised and possibly a more effective dose distribution. For example, where dose escalation was trialled based on a functionally-derived target volume there was a corresponding survival increase against the historical control (Table 5). This should be reviewed with the understanding that there has been substantial technological and practice refinement in glioblastoma treatment since 2005 and these changes may be due to other confounding factors like the categorisation of patients within individual studies, surgical practices, radiation therapy planning and delivery practices. There may be scope for further survival improvements with a more tailored approach to dose escalation, as boosting was done with a small number of discrete dose intervals.

Presently, research is concerned with ensuring the safety profile of dose-escalated RT for glioblastoma patients. Simultaneous-integrated boost dose prescriptions of 72–76 Gy delivered to volumes less than 100 cm$^3$, with the standard PTV receiving 60 Gy, appeared relatively well tolerated (Table 3). The omission of a CTV-PTV boost volume margin in most studies may have limited the effectiveness of dose escalation. Further research is needed to compare the intrinsic differences in the boost volumes indicated by the different imaging agents and to derive ways to account for imaging agent variation via uncertainty margin recipes.

### Organs at risk sparing

The specificity of target localisation in glioblastoma patients is directly linked to OAR sparing, particularly with inverse planning and intensity modulated radiation therapy (IMRT) delivery. Dose escalation with simultaneous-integrated boost techniques was common in the studies. Switching to IMRT delivery created OAR sparing capacity [41, 61]. However, current segmenting guidelines are based on anatomical imaging and thus inherently have to use relatively large isotropic expansions [89, 90]. Berberat and colleagues [69] demonstrated the feasibility of using functional imaging to map the white matter tracks in the brain and incorporated this information when deciding on target volumes. This mapping resulted in a 15% reduction in the PTV. This smaller PTV will most likely result in increased OAR sparing and increased potential for dose escalation via isotoxic planning. Both Wang [70] and Altabella [72] also used diffusion tensor imaging magnetic resonance imaging (DTI MRI) to map white matter tracks, but instead of using the information for target localisation, information was used as an IMRT OAR optimisation structure. A limitation with NM approaches to FIGR, is the lack of information regarding OAR functionality that can be incorporated into the RT plan.

### Volume comparisons and radiomics

Volume comparison studies were the most prevalent study type (n = 21). Volumes were compared using absolute comparisons (mean/median), dice scores, Hausdorff distance metrics and radiomic voxel comparisons. The variation in data (Table 2), highlighted the need for volume comparison study guidelines. For example, two of the five studies that altered treated target volumes, based on FIGR, did not report the treated CTV size [43, 46].

A current gap in the literature is the unclear relationship between volumes outlined with different functional imaging agents for FIGR. None of the studies compared target volumes based on different functional scan information. The similarity between targets based on fluorine-18-fluoroethyltyrosine positron emission tomography ($^{18}$F-FET PET) and carbon-11-methionine positron emission tomography ($^{11}$C-MET PET) is well established but information about the crossover to other types of functional scan volumes is not present in the literature.

The value of recurrence analysis in volume comparison studies cannot be underestimated as they indicated where tumour volume was either missed or did not fully respond to treatment. However, the varied terminology used in the studies to describe recurrence location in relation to the outlined volumes makes interpretation and utilisation challenging. Despite this, there was a trend to suggest that a combined approach to target voluming would improve target sensitivity (Table 4).

Volume comparison studies that report survival are key to assessing the benefits of FIGR and critical to any practice reform. The methodologies used in the studies that implemented FIGR were heterogeneous, however, survival outcomes were reported in a uniform way. The improvements in survival with FIGR (n = 10) were modest, yet consistent (Tables 3 and 5). Further improvements may be possible with optimised dose escalation and outlining.

The radiomics studies presented a pathway to incorporate functional imaging data from diverse imaging techniques with a unified approach and could address limitations associated with each imaging technology.

### Limitations and recommendations

Our study has several limitations. Variation in patient cohorts between studies should be acknowledged. Factors that influence outcomes, such as patient demographics, extent of surgery, MGMT status, chemotherapy protocol and RT treatment delivery were not clearly reported across all studies. It is not possible to verify the diagnosis and classification processes that led to patients being diagnosed with glioblastoma and thus meeting inclusion criteria for individual studies. The inclusion of published conference articles resulted in some studies having limited background detail to support data extraction.

Carrying out this review highlighted key recommendations for future FIGR studies. Implementation protocols should be published. Guideline development and implementation is needed for RT volume comparison and recurrence studies. Further, there is a need for prospective volume comparison studies between different FIGR intervention agents with radiomic analyses.

## Conclusion

Functional image-guided radiotherapy is not currently standard practice for glioblastoma patients due to the lack of conclusive Phase III evidence. However, there are many variations possible when implementing FIGR and this makes assessing competing methodologies difficult. This review highlights the different approaches to FIGR for glioblastoma patients and relevant successes.

A three-pronged approach to FIGR for the RT treatment of patients with glioblastoma is recommended with optimised target voluming, dose prescription and OAR sparing. There is a need for a more structured approach to the testing and implementation of competing methodologies, with practical recommendations to account for the variations in available technology.

## Supplementary information

**Additional file 1.** A Database Search Strategy.

## Data Availability

The datasets supporting the conclusions of this article are included within the article.

## Abbreviations

$^{11}$C-AMT PET: Carbon-11-alpha-methyltryptophan positron emission tomography
$^{11}$C-MET PET: Carbon-11-methionine positron emission tomography
$^{18}$F-FAP: Fluorine-18-fibroblast activating protein positron emission tomography
$^{18}$F-FDG PET: Fluorine-18-fluorodeoxyglucose positron emission tomography
$^{18}$F-FDOPA PET: Fluorine-18-fluorodihydroxyphenylalnine positron emission tomography
$^{18}$F-FET PET: Fluorine-18-fluoroethyltyrosine positron emission tomography
BOLD MRI: Blood oxygenation level dependent magnetic resonance imaging
CT: Computed tomography
CTV: Clinical target volume
DCE MRI: Dynamic contrast enhanced magnetic resonance imaging
DTI MRI: Diffusion tensor imaging magnetic resonance imaging
DTT MRI: Diffusion tensor tractography magnetic resonance imaging
DW MRI: Diffusion weighted magnetic resonance imaging
EORTC: European organization for research and treatment of cancer
FIGR: Functional image-guided radiotherapy
GTV: Gross tumour volume
ICRU: International commission on radiotherapy units
IGRT: Image-guided radiation therapy
IMRT: Intensity modulated radiation therapy
IDH: Isocitrate dehydrogenase
MGMT: O-6-methylguanine-DNA methyltransferase
MRI: Magnetic resonance imaging
MRSI: Magnetic resonance spectroscopic imaging
NM: Nuclear medicine
NOS: Newcastle–Ottawa scale
OAR: Organ at risk
PET: Positron emission tomography
PRISMA: Preferred reporting items for systematic reviews and meta-analyses
PTV: Planning target volume
PWI MRI: Perfusion weighted imaging magnetic resonance imaging
RT: Radiation therapy
RTOG: Radiotherapy and oncology group
TIDieR: Template for intervention description and replication
TMZ: Temozolomide
US: United States
